# Acid-dependent Interleukin-1 (IL-1) Cleavage Limits Available Pro-IL-1β for Caspase-1 Cleavage*

**DOI:** 10.1074/jbc.M115.667162

**Published:** 2015-08-31

**Authors:** Michelle E. Edye, David Brough, Stuart M. Allan

**Affiliations:** From the Faculty of Life Sciences, University of Manchester, Manchester M13 9PT, United Kingdom

**Keywords:** acidosis, caspase 1 (CASP1), inflammation, innate immunity, interleukin 1 (IL-1), cathepsin D, interleukin 1 receptor type II (IL-1R2)

## Abstract

Noncommunicable diseases such as cardiovascular disease (stroke and heart attack), cancer, chronic respiratory disease, and diabetes are a leading cause of death and disability worldwide and are worsened by inflammation. IL-1 is a driver of inflammation and implicated in many noncommunicable diseases. Acidosis is also a key feature of the inflammatory microenvironment; therefore it is vital to explore IL-1 signaling under acidic conditions. A HEK-IL-1 reporter assay and brain endothelial cell line were used to explore activity of mature IL-1α and IL-1β at pH 7.4 and pH 6.2, an acidic pH that can be reached under inflammatory or ischemic conditions, alongside cathepsin D-cleaved 20-kDa IL-1β produced under acidic conditions. We report that mature IL-1 signaling at IL-1 receptor type 1 (IL-1R1) is maintained at pH 6.2, but the activity of the decoy receptor, IL-1R2, is reduced. Additionally, cathepsin D-cleaved 20-kDa IL-1β was minimally active at IL-1R1 and was not further cleaved to highly active 17-kDa IL-1β. Therefore formation of the 20-kDa form of IL-1β may prevent the generation of mature bioactive IL-1β and thus may limit inflammation.

## Teaser

**Background:** IL-1β is an inflammatory cytokine that is activated by cleavage of an inactive precursor. Multiple proteases can cleave IL-1β, but their contribution under disease conditions is unknown.

**Results:** Cathepsin D cleaved IL-1β to a less active form than caspase-1.

**Conclusion:** During disease relevant acidic conditions, cathepsin D cleavage of IL-1β reduces IL-1β activity.

**Significance:** Cathepsin D cleaved IL-1β may limit IL-1-dependent inflammation.

## Introduction

Inflammation worsens multiple noncommunicable diseases such as stroke, heart attack, diabetes, and cancer (1–3), which are the greatest cause of mortality worldwide and a key priority for ongoing research (4). IL-1α and IL-1β are pro-inflammatory cytokines and key drivers of the inflammation observed in noncommunicable diseases. Much of the work on the mechanisms of IL-1 production and signaling has been performed on cells cultured under standard culture conditions, not relevant to disease.

Disease is associated with a change in the local environment. Following a disruption in blood flow, for example during stroke or in the metabolically active environment of a proliferating tumor or epileptic brain undergoing a seizure, a drop in pH is observed (5–7). Inflammation is also associated with a decrease in pH. As inflammation progresses, there is an increase in infiltrating immune cells, an increase in glycolysis, and subsequent elevation in lactic acid and drop in pH (8, 9). Whether this acidosis affects the inflammatory signaling observed in disease is unknown.

IL-1α and β are potent inducers of inflammation; thus their activation and release are tightly regulated. Both IL-1α and β are synthesized as pro forms, and although initial studies suggested that pro-IL-1α possessed activity at its receptor (IL-1 receptor type 1; IL-1R1),^2^ it is now known that both pro-IL-1α and pro-IL-1β require a second activating step to induce their cleavage to exert maximal activity at IL-1R1 (10). Pro-IL-1α is cleaved via calcium-dependent cysteine proteases of the calpain family to produce 17-kDa IL-1α (10). Cleavage of pro-IL-1β is typically performed by the cysteine protease caspase-1 to produce 17-kDa mature IL-1β. Caspase-1 itself requires activation via association with a protein complex called an inflammasome before it can cleave pro-IL-1β (11).

In addition to regulation of IL-1α and β at the level of processing, a decoy receptor (IL-1R type II; IL-1R2), endogenous receptor antagonist (IL-1Ra), and intracellular negative regulators (TIR8/SIGIRR or micro-RNAs) exist to control IL-1 activity (12). Mutations in these regulators could result in excessive IL-1 signaling, contributing to the development or exacerbation of inflammatory diseases. Indeed, a gain of function mutation in the NLRP3 (NACHT, LRR, and PYD domain-containing protein 3) inflammasome results in the autoinflammatory diseases cryopyrin-associated periodic syndromes (13), and the protective role of IL-1R2 has been shown in a mouse collagen-induced arthritis model where IL-1R2 KO mice have increased arthritis severity (14).

Because of the established role of IL-1 in disease, it has been targeted therapeutically (15). However, targeting IL-1β activation with caspase-1 inhibitors has not always been effective in preventing IL-1β signaling, and recent evidence has emerged for a role of caspase-1-independent IL-1β processing in disease (16, 17). Although much work has been done on caspase-1-dependent IL-1β processing, research into mechanisms of caspase-1-independent IL-1β processing is less studied. Under acidic conditions, caspase-1-independent 20-kDa IL-1β is produced (18). The 20-kDa form of IL-1β has been suggested to be 5-fold less active than mature 17-kDa IL-1β (19). However, the activity of 20-kDa IL-1β has not been investigated under the acidic conditions in which it is produced. Additionally, the activity of mature IL-1 has not been studied under acidic conditions, nor have the interactions between these forms of IL-1. HEK-Blue IL-1β reporter cells and a brain endothelial cell line were used to explore the role of 20-kDa IL-1β in IL-1 signaling pathways. The cells were used to determine the activity of 20-kDa IL-1β and investigate its signaling alongside mature 17-kDa IL-1 α and β at pH 6.2. Our data suggest that 20-kDa IL-1β is a negative regulator of IL-1β that limits the amount of pro-IL-1β available for caspase-1 cleavage.

## Experimental Procedures

### 

#### 

##### IL-1 Activity Assay

HEK-Blue IL-1β cells (InvivoGen) were used to determine IL-1β activity. Cells were cultured in selective media (DMEM (Sigma), 10% FBS (Life Technologies), 100 units/ml penicillin, 100 μg/ml streptomycin (Sigma), 100 μg/ml Normocin (InvivoGen), 100 μg/ml Zeocin (InvivoGen), 200 μg/ml HygroGold (InvivoGen)) to express IL-1R1. Cells were seeded at 3.3 × 10^5^ cells per ml in a 96-well plate (∼50,000 cells/well). Treatments were made up in HEPES-buffered salt solution (145 mm NaCl, 2.5 mm KCl, 1 mm MgCl_2_, 1.8 mm CaCl_2_, 20 mm HEPES, 10 mm glucose, 0.01% BSA as described previously,) (18) buffered to the indicated pH with 1 m NaOH and incubated with HEK-IL-1β reporter cells overnight. In these cells, activation of IL-1R1 results in NF-κB-driven expression of secreted embryonic alkaline phosphatase, which causes a color change when incubated with QUANTI-Blue. Absorbance was measured at 630 nm on a Synergy HT plate reader (BioTek).

Little reporter activity was observed with cells incubated with IL-1 at pH 6.2 overnight (data not shown), but in line with a transient reduction in pH to model acute acidosis during inflammation, cells were treated at pH 6.2 for up to 60 min before the medium was changed to DMEM 100 units/ml penicillin 100 μg/ml streptomycin overnight. Control cells treated at pH 7.4 alongside pH 6.2-treated cells also underwent a media change to DMEM, 100 units/ml penicillin, 100 μg/ml streptomycin overnight. When a media change was required, cells were seeded at least 4 h prior to use. IL-1R2 and IL-1R accessory protein (IL-1RAcP) were purchased from R & D Systems, and IL-1Ra (Kineret) was obtained from the hospital pharmacy and PBS was purchased from Sigma.

##### bEnd.5 Cells

Brain endothelial cell line bEnd.5 (from Health Protection Agency Culture Collections) was cultured in DMEM (Sigma) supplemented with 10% FBS (Life Technologies), 100 units/ml penicillin, and 100 μg/ml streptomycin (Sigma). Cells were seeded at 2 × 10^5^ cells per ml in a 96-well plate overnight. Treatments were made up in reducing media (100 mm NaCl, 50 mm HEPES, 10 mm DTT, 1 mm EDTA, 10% glycerol, 1% CHAPS) at pH 6.2. Pro-IL-1β (100 ng/ml; Sino Biological Inc.) was incubated with 100 units/ml caspase-1 (Calbiochem) and/or 1 units/ml cathepsin D (Calbiochem) for 8 h and then incubated with cells for 60 min before being replaced with DMEM, 100 units/ml penicillin, 100 μg/ml streptomycin for a further 23 h. Supernatants were collected on ice and stored at −20 °C until required.

##### Cathepsin D Cleavage Assay

Human pro-IL-1β (100 ng/ml; Sino Biological Inc.) was incubated with human cathepsin D (1 units/ml; Calbiochem) in HEPES-buffered salt solution at the indicated pH for 60 min unless stated otherwise.

##### Caspase-1 Cleavage Assay

To ensure all pro-IL-1β had been cleaved to the 20-kDa IL-1β from prior to the addition of caspase-1, pro-IL-1β was incubated with cathepsin D for 8 h. Caspase-1 (100 units/ml; catalogue number 218783; Calbiochem) was incubated overnight with 20-kDa IL-1β, pro-IL-1β, or vehicle in reducing media (see above) buffered to indicated pH with 1 m NaOH.

##### Western Blot

Samples were denatured at 95 °C for 5 min prior to separation on a 12% acrylamide gel and transfer to a nitrocellulose membrane on a Trans-Blot SD Semi-Dry Transfer Cell (Bio-Rad). Membranes were blocked with 5% milk before incubation with goat anti-human IL-1β primary antibody (0.1 μg/ml; R & D Systems; AF-201) overnight at 4 °C. Membranes were washed, incubated with HRP-conjugated rabbit anti-goat secondary antibody (1/1000; Dako; P0449), and then enhanced chemiluminescence (Amersham Biosciences). Film was processed on a JP-33 automatic film processor.

##### ELISA

Supernatants were analyzed for IL-6 and CXCL1 using specific ELISA kits (R & D Systems).

##### Statistical Analysis

Statistical analysis was performed using GraphPad Prism v6. For comparisons between multiple groups, a one-way analysis of variance (ANOVA) with a Bonferroni multiple comparison post hoc test was used. When unequal variance was observed (S.E. proportional to the mean value), data were log transformed prior to statistical analysis. For comparisons between time course data, a two-way ANOVA with Bonferroni multiple comparison post hoc test was used. For competition experiments, data were expressed as relative activity/release compared with IL-1-induced activity/release which was normalized to 100%. Because this removes the variance in the control group, for normalized results, a one-sample *t* test against a hypothetical value (100%) with a Bonferroni multiple comparison post hoc test was used. The data are expressed as means ± S.E., and *p* ≤ 0.05 was considered significant.

## Results

### 

#### 

##### Acidic pH Does Not Affect the Activity of IL-1 at IL-1R1

**FIGURE 1.**
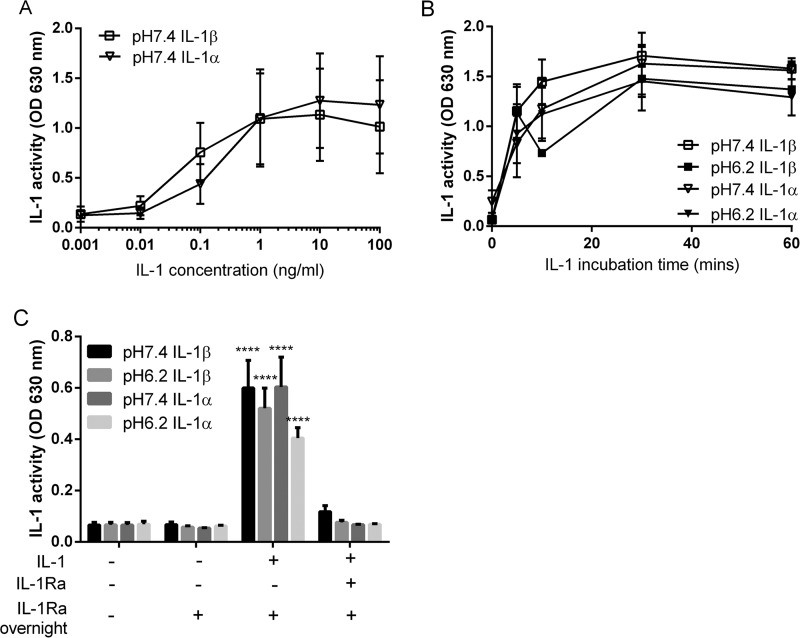
**17-kDa IL-1α and IL-1β activity at IL-1R1 at pH 7. 4 and pH 6.2.** A HEK-IL-1 reporter assay (InvivoGen) was used to determine the activity of IL-1, expressed as optical density at 630 nm. *A*, serial dilutions of IL-1α and β at pH 7.4 were incubated with HEK-IL-1 cells overnight before IL-1 activity was recorded at 630 nm. *B*, 10 ng/ml IL-1α or β was incubated with HEK-IL-1 cells for 0–60 min at pH 7.4 or 6.2 prior to a further incubation with DMEM, 100 units/ml penicillin, 100 μg/ml streptomycin overnight before IL-1 activity was recorded at 630 nm. *C*, 1 μg/ml IL-1Ra (or 0.1% BSA in PBS vehicle) was incubated with the cells overnight following the initial 60-min incubation with 10 ng/ml IL-1 as in *B*. IL-1Ra was also included for the entire duration of the experiment as a positive control. The data are expressed as means ± S.E., *n* = 3–4. Statistical analysis for *A* and *B* did not find any significance between groups using a two-way ANOVA with Bonferroni's post hoc test. Statistical analysis for *C* was performed with a one-way ANOVA followed by Bonferroni's post hoc test following a log transformation to adjust for unequal variance. No significant difference was detected in IL-1 activity between pH 7.4 and pH 6.2 with 60 min IL-1α or β treatment plus IL-1Ra overnight. No significant difference was observed between IL-1 plus IL-1Ra for the duration of the experiment and the control (IL-1Ra overnight). ****, *p* < 0.0001 compared with control (IL-1Ra overnight).

Under inflammatory or ischemic conditions, local pH can drop as low as pH 6.2 (5). A HEK-Blue IL-1β reporter assay that measures NF-κB-mediated secreted embryonic alkaline phosphatase release after IL-1R1 activation by IL-1β was used to measure the activity of IL-1 at neutral and acidic pH. As expected, at pH 7.4, activity increased with increasing IL-1β concentrations (Fig. 1*A*, *squares*). Activity also increased with increasing IL-1α concentrations at pH 7.4 (Fig. 1*A*, *triangles*), suggesting that this assay can additionally be used to measure IL-1α activity. When cells were incubated with 10 ng/ml IL-1 at pH 6.2 for times up to 60 min, to model acute acidosis, IL-1β- and IL-1α-induced activity was not significantly altered when measured 16–18 h later compared with pH 7.4 (Fig. 1*B*). To confirm that the IL-1 activity recorded was due to actions of IL-1 during the initial 60-min incubation at pH 6.2 or 7.4 and not the activity of residual IL-1 remaining during the subsequent overnight incubation, cells were treated with IL-1 for 60 min as in Fig. 1*B* but replaced with media containing IL-1Ra for the subsequent overnight incubation to block any further activation of IL-1R1. Cells treated in this way continued to produce IL-1-induced secreted embryonic alkaline phosphatase activity, suggesting that a 60-min incubation is sufficient to induce activation of IL-1R1 and subsequent downstream activation of NF-κB signaling. Treatment with IL-1Ra for the duration of the initial IL-1 treatment and subsequent overnight incubation prevented any IL-1-induced activity, confirming that the dose used was sufficient to block IL-1 activity at IL-1R1 in this model (Fig. 1*C*). These data confirm that IL-1 signaling was not affected by acidic pH.

##### Cathepsin D Cleaves Pro-IL-1β to a 20-kDa IL-1β Species

**FIGURE 2.**
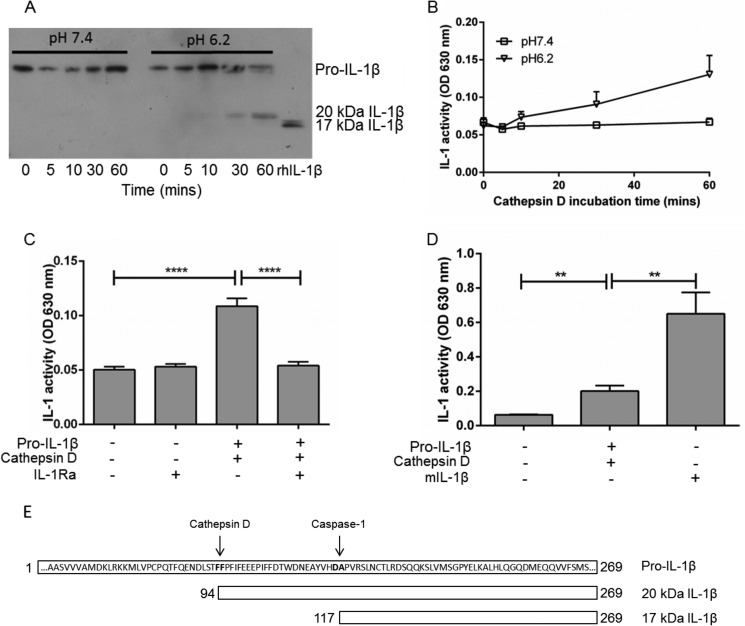
**Production and activity of cathepsin d-cleaved 20-kDa IL-1β.** Recombinant pro-IL-1β (100 ng/ml) was incubated with 1 units/ml cathepsin D for 0–60 min at pH 7.4 or 6.2. *A*, samples were processed by Western blot analysis alongside recombinant human 17-kDa IL-1β (rhIL-1β) in the final lane. *B*, these same samples were also incubated with HEK-IL-1 reporter cells. Samples from 0–60 min pro-IL-1β plus cathepsin D incubation were added to HEK-IL-1 cells for 60 min before being replaced with fresh media overnight. *Squares* represent samples of pro-IL-1β incubated with cathepsin D for 0–60 min at pH 7.4; *triangles* represent pH 6.2. *C*, samples from 60 min pro-IL-1β plus cathepsin D incubation were added to HEK-IL-1 cells ± 1 μg/ml IL-1Ra for 60 min before being replaced with fresh media overnight. *D*, samples from 60 min pro-IL-1β plus cathepsin D incubation were added to HEK-IL-1 cells and compared alongside mature 17-kDa IL-1β for 60 min before being replaced with fresh media overnight. *E*, schematic of pro-IL-1β and putative cathepsin D (29) and caspase-1 cleavage sites adapted from Hazuda *et al.* (19). Western blot is representative of *n* = 3; graphical data are expressed as means ± S.E., *n* = 3–4. Statistical analysis was performed with a one-way ANOVA with Bonferroni's multiple comparison test. Significance was determined as *p* ≤ 0.05. **, *p* < 0.01; ****, *p* < 0.0001.

We have previously reported that at pH 6.2, caspase-1-independent 20-kDa IL-1β is released from mixed glia and human macrophages (18). Here we confirmed that recombinant cathepsin D was able to cleave pro-IL-1β into a 20-kDa form under acidic conditions (Fig. 2*A*) and that this displayed activity at IL-1R1 (Fig. 2, *B* and *C*), but this was reduced compared with mature IL-1β (Fig. 2*D*). This is in line with previous literature showing that HIV protease-cleaved 20-kDa IL-1β is ∼5-fold less active than mature IL-1β at neutral pH (19).

To confirm that any activity shown with the 20-kDa IL-1β was acting through IL-1R1, we incubated HEK-IL-1 reporter cells with the 20-kDa IL-1β produced from 60-min incubation of pro-IL-1β and cathepsin D ± IL-1Ra (Fig. 2*C*). 20-kDa IL-1β activity was completely abolished on coincubation with IL-1Ra, confirming that 20-kDa IL-1β was acting at IL-1R1.

##### 20-kDa IL-1β Does Not Affect Mature IL-1α or IL-1β Signaling at IL-1R1

**FIGURE 3.**
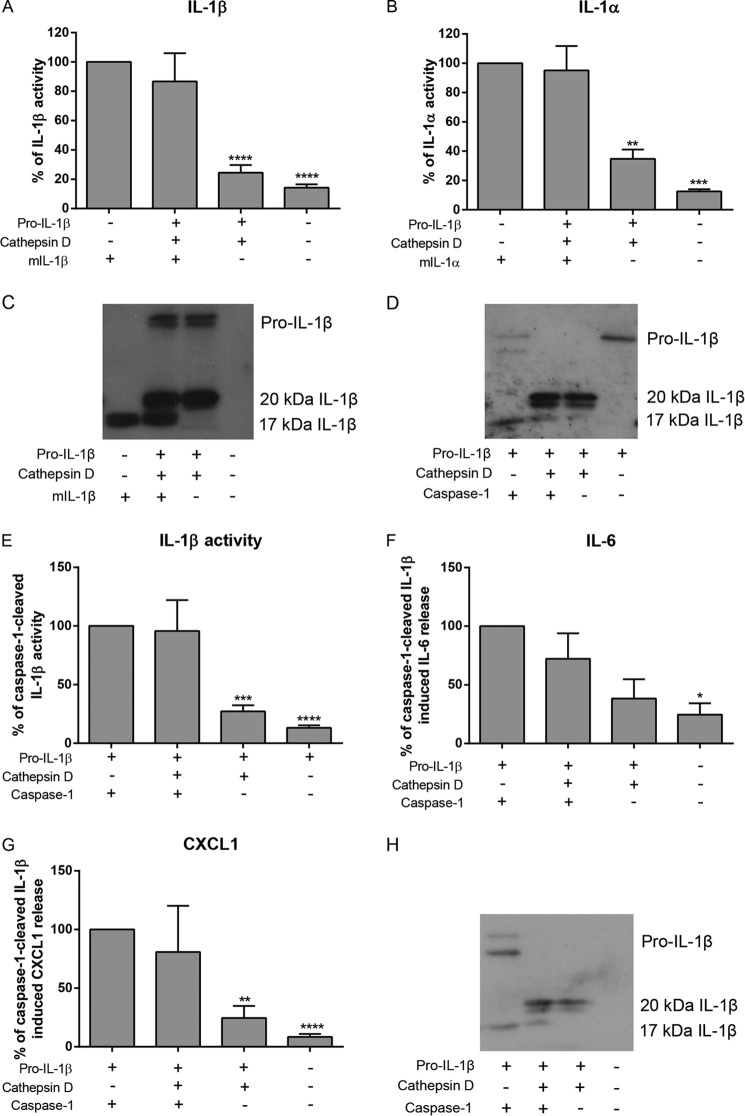
**IL-1 activity in the presence of 20-kDa IL-1β.** *A* and *B*, 100 ng/ml pro-IL-1β was incubated with 1 units/ml cathepsin D for 60 min to produce 20*-*kDa IL-1β before this was added to HEK-IL-1 cells with 10 ng/ml recombinant mature IL-1β (*mIL-1*β; *A*) or IL-1α (*mIL-1*α; *B*) for 60 min. This was then replaced with fresh media overnight. *C*, pro-IL-1β cleavage following 60 min of incubation with 1 units/ml cathepsin D was confirmed by Western blot alongside 10 ng/ml recombinant mature IL-1β. *D*, 100 ng/ml pro IL-1β was incubated with caspase-1 (100 units/ml), cathepsin D (1 units/ml), or both overnight at pH 6.2, and cleavage was confirmed by Western blot. *E*, the resulting samples were added to HEK-IL-1 cells for 60 min before being replaced with fresh media overnight. Samples from an 8-h incubation of 100 ng/ml pro-IL-1β with 100 units/ml caspase-1 and/or 1 units/ml cathepsin D were added to brain endothelial cells (bEnd.5) for 60 min before being replaced with fresh media overnight. *F* and *G*, levels of IL-6 (*F*) and CXCL1 (*G*) released were quantified by ELISA. *H*, pro-IL-1β cleavage during the 8-h incubation with caspase-1 and cathepsin D was confirmed by Western blot. Western blots are representative of *n* = 3. Data are presented relative to activity induced by mature 17-kDa IL-1 that was normalized to 100%. Data are the means ± S.E. *n* = 3–5. Statistical analysis was performed with a one-sample *t* test against a hypothetical value (100%) with a Bonferroni multiple comparison post hoc test. *p* ≤ 0.05 was considered significant. *, *p* < 0.05; **, *p* < 0.01; ***, *p* < 0.001; ****, *p* < 0.0001.

To understand the relevance of 20-kDa IL-1β in IL-1 signaling under disease relevant acidic conditions, we investigated whether 20-kDa IL-1β would affect active 17-kDa IL-1 signaling at pH 6.2. 20-kDa IL-1β (generated by the incubation of 100 ng/ml pro-IL-1β and 1 units/ml cathepsin D at pH 6.2) was added to HEK-IL-1 cells with 10 ng/ml mature 17-kDa IL-1β (Fig. 3*A*) or IL-1α (Fig. 3*B*) at pH 6.2 for 60 min before being replaced with serum-free media overnight. The activity induced by 17-kDa IL-1 alone was normalized to 100%, and the other treatments were expressed relative to this. Under these conditions 20-kDa IL-1β did not alter the activity of 17-kDa IL-1β or IL-1α at IL-1R1. To more closely mimic a potential inflammatory scenario, cathepsin D and caspase-1 were incubated together with pro-IL-1β at pH 6.2. This resulted in complete processing of pro-IL-1β producing predominantly 20-kDa IL-1β with a small amount of 17-kDa IL-1β (Fig. 3*D*, *lane 2*). When this was added to HEK-IL-1 cells, there was still no significant change in activity from caspase-1 cleaved 17-kDa IL-1β alone (Fig. 3*E*), again suggesting that the 20-kDa form is not able to influence the 17-kDa species at IL-1R1. The 20-kDa form may, however, act to remove available pro-IL-1β for further caspase-1 cleavage (see below).

To investigate 20-kDa IL-1β signaling in brain endothelial cells, which are known to respond to IL-1β and contribute to the inflammatory response in central nervous system disorders (20), samples from a 8-h incubation of pro-IL-1β with cathepsin D and/or caspase-1 were added to the mouse brain endothelial cell line, bEnd.5, for 60 min before being replaced with serum free media for a further 23 h. Incubation of pro-IL-1β with caspase-1 and cathepsin D predominantly resulted in the production of 20-kDa IL-1β with some 17-kDa IL-1β (Fig. 3*H*). IL-1-mediated activity (as measured by IL-6 and CXCL1 release) with the caspase-1-cleaved plus cathepsin D-cleaved 17-kDa and 20-kDa IL-1β was not significantly different from caspase-1-cleaved 17-kDa IL-1β alone (Fig. 3, *F* and *G*), again suggesting that 17-kDa IL-1β signaling at IL-1R1 is not directly influenced by the 20-kDa form.

##### IL-1R2 Decoy Activity Is Reduced at pH 6.2

**FIGURE 4.**
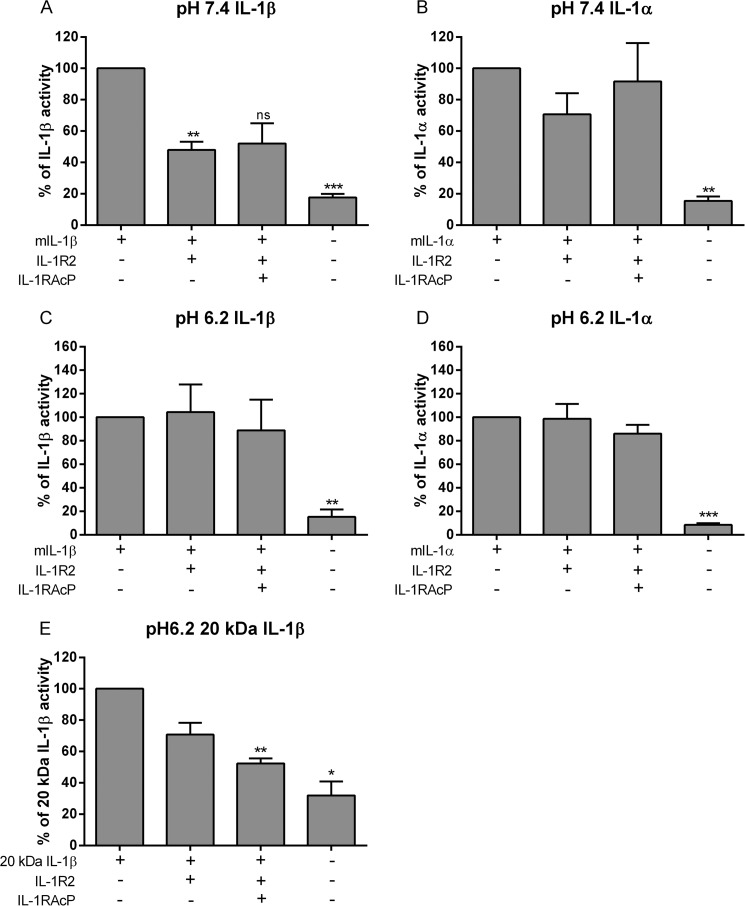
**Effect of IL-1R2 on IL-1 activity at pH 7. 4 and pH 6.2.** *A–D*, 10 ng/ml 17-kDa mature IL-1β (*mIL-1*β; *A* and *C*), 10 ng/ml 17-kDa mature IL-1α (*mIL-1*α; *B* and *D*) or the product of a 60-min incubation of 100 ng/ml pro-IL-1β with 1 units/ml cathepsin D to produce 20-kDa IL-1β (*E*) was added to HEK-IL-1 cells at the indicated pH with 2.5 μg/ml IL-1R2, 2 μg/ml IL-1RAcP, or vehicle (PBS for IL-1, IL-1RAcP, cathepsin D, or 0.1% BSA in PBS for IL-1R2) for 60 min before being replaced with fresh media overnight. Data are presented relative to activity induced by IL-1 alone (17-kDa IL-1β in *A* and *C*; 17-kDa IL-1α in *B* and *D*; and 20-kDa IL-1β in *E*) that was normalized to 100% + S.E., *n* = 3–6. Statistical analysis was performed with a one-sample *t* test against a hypothetical value (100%) with Bonferroni's multiple comparison test. Significance was determined as *p* ≤ 0.05. *, *p* < 0.05; **, *p* < 0.01; ***, *p* < 0.001.

The decoy receptor IL-1R2 binds IL-1 with different affinities, binding IL-1β with high affinity but requiring the presence of IL-1RAcP for efficient IL-1α binding (12, 21). Whether IL-1R2 signaling is altered under disease-relevant acidic conditions is unknown, as is the ability of this receptor to bind 20-kDa IL-1β. Using the HEK-IL-1 reporter assay, IL-1β activity was reduced with soluble IL-1R2 at pH 7.4 (Fig. 4*A*) but not pH 6.2 (Fig. 4*C*). Under these conditions, IL-1R2 did not affect IL-1α signaling alone or in combination with IL-1RAcP at pH 7.4 or pH 6.2 (Fig. 4, *B* and *D*). At pH 6.2, 20-kDa IL-1β signaling was reduced with IL-1R2 and IL-1RAcP combined (Fig. 4*E*). These data suggest that IL-1R2 may only have a minor role in the regulation of mature IL-1 under acidic conditions.

##### 20-kDa IL-1β Is Not Further Cleaved by Caspase-1

**FIGURE 5.**
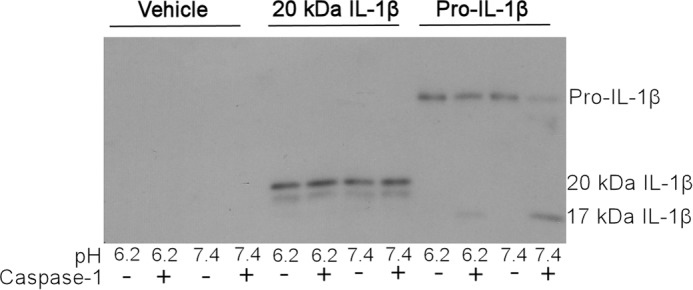
**Western blot analysis of caspase-1 cleavage.** Vehicle (PBS), 20-kDa IL-1β (produced from 8-h incubation of 100 ng/ml pro-IL-1β with 1 units/ml cathepsin D in reducing media at pH 6.2), or 100 ng/ml pro-IL-1β was incubated with 100 units/ml caspase-1 or vehicle (PBS) overnight in reducing media buffered to indicated pH with 1 m NaOH. Samples were collected on ice and analyzed by Western blot. The image is representative of *n* = 3.

To assess whether 20-kDa IL-1β is a temporary holding state before further cleavage by caspase-1, 20-kDa IL-1β was incubated with recombinant caspase-1 at pH 6.2 and 7.4. Caspase-1 was able to cleave pro-IL-1β into 17-kDa mature IL-1β at pH 7.4 and to a lesser extent at pH 6.2, but it did not cleave 20-kDa IL-1β (produced from complete cleavage of pro-IL-1β following 8 h of incubation with 1 units/ml cathepsin D) into 17-kDa IL-1β at either pH (Fig. 5). These data suggest that the formation of 20-kDa IL-1β may act to limit IL-1β signaling by reducing the pro-IL-1β pool available for caspase-1 cleavage.

## Discussion

Acidosis is a key feature of disease, and yet few researchers take this into account when designing *in vitro* studies. Our findings show that although mature 17-kDa IL-1α and β signaling at IL-1R1 is not altered at low pH or in the presence of cathepsin D-cleaved 20-kDa IL-1β, production of 20-kDa IL-1β may act to limit IL-1β signaling by reducing the pool of pro-IL-1β available for caspase-1 cleavage, thus preventing the appearance of further mature 17-kDa IL-1β. We also show that IL-1R2 was able to reduce IL-1β signaling at IL-1R1 at pH 7.4 but not at pH 6.2; thus the production of 20-kDa IL-1β may partly compensate for this lack of IL-1R2-mediated control at sites of inflammation where there is reduced pH.

Because 20-kDa IL-1β binds the same receptor as 17-kDa IL-1 but only acts as a partial agonist (Fig. 2), we questioned whether 20-kDa IL-1β would compete with 17-kDa IL-1 and therefore reduce activity at IL-1R1. 20-kDa IL-1β did not have any significant effect on mature IL-1 signaling in our model, even when produced in excess compared with 17-kDa IL-1β. This may be due to the sensitivity of cells to respond to mature IL-1 and the excess of IL-1R1 receptors on their surface. It is due to this excess of receptors that IL-1Ra is produced at up to 1000-fold the amount of IL-1 to effectively inhibit IL-1 (22). The absolute amounts of 20-kDa IL-1β produced *in vivo* would need to be analyzed to determine whether 20-kDa IL-1β could act as an antagonist like IL-1Ra; however, our data suggest that its regulatory role is more likely to be in removing pro-IL-1β to prevent further cleavage by caspase-1.

There was a significant reduction in IL-1β activity with IL-1R2 at pH 7.4 but not at pH 6.2; thus our data suggest that IL-1R2 is less effective at blocking IL-1 activity at low pH. A significant reduction in 20-kDa IL-1β activity was observed with IL-1R2 and IL-1RAcP at pH 6.2, and this may in part be due to the lower activity of 20-kDa IL-1β requiring less binding by IL-1R2 to induce an observable effect. However, once 20-kDa IL-1β has been formed, it has performed its regulatory role in preventing the formation of mature 17-kDa IL-1β. Thus binding IL-1R2/IL-1RAcP would further help reduce IL-1-mediated inflammation because it would block any minor signaling induced by 20-kDa IL-1β.

Activation of caspase-1 can induce pyroptosis, resulting in a loss of plasma membrane integrity and release of intracellular components. Nearly 50% of pro-IL-1β produced in response to nigericin is released from bone marrow-derived macrophages without any further cleavage (23). Although pro-IL-1β is not active, it is now known that inflammasome components and mature caspase-1 can be released from cells and cleave extracellular pro-IL-1β into its 17-kDa active form (23). Unlike IL-1R1, IL-1R2 is able to bind pro-IL-1β, thereby preventing any subsequent cleavage to mature IL-1β. If IL-1R2 is unable to do this at low pH, cathepsin D-dependent cleavage of pro-IL-1β to 20-kDa IL-1β could fulfill this IL-1-limiting role and prevent the spread of inflammatory signaling.

Cathepsin D is an aspartyl protease that resides in the lysosome and is maximally active at low pH. The cellular location of cathepsin D cleavage of pro-IL-β (*i.e.* within lysosomes or extracellularly following pro-IL-1β release) remains to be elucidated (18, 24).

In addition to caspase-1, multiple other proteases are now known to cleave pro-IL-1β (25). Proteinase 3, a neutrophil protease, cleaves pro-IL-1β near to the caspase-1 binding site to produce an active protein (26), and inhibiting this protease is protective in a mouse model of arthritis (17). These alternatively cleaved IL-1β forms appear to be pro-inflammatory, and therefore blocking their activity could prove beneficial in disease. Moving away from the caspase-1 cleavage side toward the N terminus results in increasingly larger and less active forms of IL-1β (19), with Black *et al.* (27) suggesting no further activity observed at greater than 18.4 kDa in size. Whether other larger alternatively cleaved forms of IL-1β are protected from further cleavage to active 17 kDa and could therefore represent an additional negative regulation of IL-1β signaling is yet to be elucidated.

Lactic acid is able to induce the release of 20-kDa IL-1β (18) and has also been suggested to exert anti-inflammatory effects through a reduction of LPS-induced NF-κB signaling (28). Mature 17-kDa IL-1β signaling through IL-1R1 also activates NF-κB-induced expression of pro-inflammatory cytokines. Thus it would follow that a reduction in mature 17-kDa IL-1β caused by lactic acid-mediated production of 20-kDa IL-1β, would lead to a reduction in IL-1β signaling at IL-1R1 and therefore a subsequent reduction in IL-1-induced pro-inflammatory cytokines. Alternatively Rajamäki *et al.* (9) show that acidosis can activate the inflammasome, inducing mature 17-kDa IL-1β release. These differences may be due to the type of acid used because this has previously been shown to determine whether a pro- or anti-inflammatory effect is observed, because lactic acid exerts effects different from those of hydrochloric acid (28).

## Conclusion

Our data suggest that the formation of 20-kDa IL-1β under acidic conditions provides negative regulation of IL-1 signaling. Cleavage of pro-IL-1β to 20-kDa IL-1β inhibits its further cleavage to the highly active 17-kDa IL-1β, thereby limiting the inflammatory cascade and dampening pro-inflammatory signaling at sites of inflammation where a reduction in pH is observed. Additional mechanisms of regulation of this system are particularly important at low pH as the ability of IL-1R2 to inhibit IL-1 signaling is reduced. The *in vitro* system used here is a valuable tool for exploring signaling pathways, and the use of acidic pH to more closely mimic the disease microenvironment is an improvement on physiological culture conditions. However, it is not a perfect model of human disease; thus further work *in vivo* and in disease tissue is required to fully understand the role of 20-kDa IL-1β.

## Author Contributions

M. E. E., D. B., and S. M. A. designed the study. M. E. E. performed and analyzed the experiments and wrote the manuscript with critical review by D. B. and S. M. A. All authors reviewed the results and approved the final version of the manuscript.

## Footnotes

2 The abbreviations used are:

IL-1R: IL-1 receptor
IL-1RAcP: interleukin-1 receptor accessory protein
ANOVA: analysis of variance.
